# Identifying priority healthcare trainings in frozen conflict situations: The case of Nagorno Karabagh

**DOI:** 10.1186/1752-1505-4-21

**Published:** 2010-12-09

**Authors:** Michael E Thompson, Alina H Dorian, Tsovinar L Harutyunyan

**Affiliations:** 1 Assistant Professor Coordinator, MSPH Program Department of Public Health Sciences, University of North Carolina at CharlotteCharlotte, NC, USA; 2 Adjunct Assistant Professor College of Health Sciences, American University of Armenia Yerevan, Armenia; 3 Assistant Professor, Community Health Sciences, UCLA School of Public Health Assistant Director, International Programs, UCLA Center for Public Health and Disasters University of California at Los Angeles Los Angeles, CA, USA; 4 PhD student College of Health and Human Services, University of North Carolina at Charlotte Charlotte, NC, USA

## Abstract

**Introduction:**

Health care in post-war situations, where the system's human and fixed capital are depleted, is challenging. The addition of a frozen conflict situation, where international recognition of boundaries and authorities are lacking, introduces further complexities.

**Case description:**

Nagorno Karabagh (NK) is an ethnically Armenian territory locked within post-Soviet Azerbaijan and one such frozen conflict situation. This article highlights the use of evidence-based practice and community engagement to determine priority areas for health care training in NK. Drawing on the precepts of APEXPH (Assessment Protocol for Excellence in Public Health) and MAPP (Mobilizing for Action through Planning and Partnerships), this first-of-its-kind assessment in NK relied on in-depth interviews and focus group discussions supplemented with expert assessments and field observations. Training options were evaluated against a series of ethical and pragmatic principles.

**Discussion and Evaluation:**

A unique factor among the ethical and pragmatic considerations when prioritizing among alternatives was NK's ambiguous political status and consequent sponsor constraints. Training priorities differed across the region and by type of provider, but consensus prioritization emerged for first aid, clinical Integrated Management of Childhood Illnesses, and Adult Disease Management. These priorities were then incorporated into the training programs funded by the sponsor.

**Conclusions:**

Programming responsive to both the evidence-base and stakeholder priorities is always desirable and provides a foundation for long-term planning and response. In frozen conflict, low resource settings, such an approach is critical to balancing the community's immediate humanitarian needs with sponsor concerns and constraints.

## Introduction

Evidence-based approaches in public health practice provide a systematic, objective framework that can inform policy and decision-making by establishing priorities that make maximal use of limited resources. Within the realm of humanitarian assistance, the evidence on how to respond to disasters has evolved: Public health specialists and Non-governmental Organizations (NGOs) have developed protocols for preparing for and managing responses to earthquakes, cyclones, natural disasters, and, sadly, endemic wars [1-4] and evidence is emerging on how best to transition from humanitarian response to development [5,6]. Little is known, however, about the added challenges of health sector development and health sector human resources management in frozen conflicts [7,8], where peace has been negotiated but international recognition of boundaries and authorities are lacking. Such is the situation found in Nagorno Karabagh (NK) [9], an ethnic Armenian territory locked within post-Soviet Azerbaijan.

**Figure 1:**
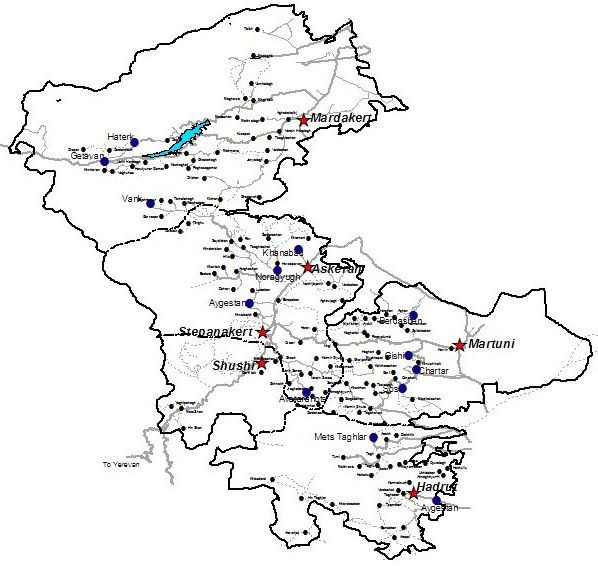
**Map of Nagorno Karabagh**. Prepared by the Acopian Center for the Environment, American University of Armenia, 2003. *Note: Stars indicate regional capitals. Circles represent cities and villages, with the circle size proportional to the population*

Nagorno Karabagh is a fertile, mountainous region located in the northeastern part of the Armenian highlands [10] [See map, Figure 1]. Part of pre-soviet Armenia, Stalin annexed NK to Azerbaijan in 1923 [11] where it functioned as a semi-autonomous Oblast, an administrative division used by the USSR to recognize where a majority of the population differed nationally or ethnically from the republic's majority, until 1988 when it declared itself independent, sparking a fierce military conflict with Azerbaijan. The conflict escalated in 1991 due to the dissolution of the Soviet Union and Armenia's active support of NK's independence movement. Fighting lasted until 1994, when a cease-fire was enacted. Although the cease-fire has held, a permanent peace has not been negotiated: the conflict has been "frozen," with little progress made in the past 15 years despite intensive efforts by the international community to foment a peace process. Consequently, NK is not internationally recognized as an independent nation [9].

The absence of international recognition presents a serious impediment to NK's recovery, as it hinders international communications, trade and foreign assistance that countries emerging from war situations typically receive [12]. Thus, NK is currently experiencing a period of relative peace, but with no diplomatic guarantees, limiting international response and making planning difficult. The conflict devastated NK's economy and resulted in many thousand deaths and over one million refugees and displaced persons [9]. NK's 2002 estimated population was 145,000, of whom over 95% are Armenians [13]. Approximately 36,000 Armenian refugees from Azerbaijan and approximately 71,000 internally displaced Armenians current live in NK [13]. The small republic has revived government services and established a functioning, albeit unrecognized, state. The *de facto *government faces many challenges to meeting the population's health and human services needs [12].

The economic and political climate in NK has created difficulties for health care delivery [14,15]. Health services delivery has been intermittently disrupted and supplies are chronically unavailable. These severe hardships negatively affect individuals' and families' health and health seeking behavior. Social and family networks, the usual safety nets for health problems and economic difficulties, are strained. Environmental conditions have deteriorated drastically, reflecting the trauma of war and the ensuing frozen conflict that followed. The resulting challenges to planning, financing, and implementing health programs is felt by specialists, humanitarian organizations, and, most acutely, the region's population. Little is presently known about the true needs or how best to respond to them.

International non-governmental organizations and the Armenian Diaspora have addressed some of NK's most urgent health challenges. However, the NK population now simultaneously suffers transition health problems such as infectious and parasitic diseases (including tuberculosis outbreaks) and conditions more typical for post-transition populations: heart diseases, cancers, and diabetes [14], often referred to as a protracted polarized epidemiologic transition [16]. Although no large-scale epidemics of communicable diseases have been reported in NK since 1988, numerous public health problems have intensified. Diarrheal diseases and acute respiratory infections (ARI) are highly prevalent in children. Childhood trauma and injuries are reportedly a significant public health problem with the main causes being fractures, burns, and landmine injuries; however, exact figures are not currently available.

Responding to the need for an integrated humanitarian support program, the United States Agency for International Development (USAID) in 2003 contracted the Fund for Armenian Relief (FAR) and the American University of Armenia's (AUA) Center for Health Services Research and Development (CHSR) to carry out the Humanitarian Assistance Project in Nagorno Karabagh (HAP-NK). The AUA CHSR implemented the health component of the program, which envisioned a combined approach of infrastructure rehabilitation paired with targeted workforce development activities. The first phase of the project (2004) consisted of parallel detailed health facility and health worker training needs assessments.

The healthcare workforce is vital to protecting and advancing health. Developing competent healthcare providers is central to achieving national and global health goals [17]. Governments are responsible for assuring the capabilities of newly entering healthcare workers into the workforce and assisting schools, universities, and training colleges to produce high quality professionals. Rapid increases in medical knowledge and changing health systems, however, make lifelong learning for health professionals equally important [17]; thus, presenting a great challenge for developing countries where many health workers are underpaid, poorly motivated, and dissatisfied [18]. This challenge is even greater for post-war situations where active military conflict depletes the system's human and fixed capital. Beyond damaging clinics, hospitals, laboratories, and health care centers, military conflicts often lead to the emigration of younger and more highly trained medical professionals, a trend that is difficult to reverse [19]. The situation is further compounded by the system's inability to provide training opportunities for healthcare providers and the pent-up "information hunger" that exists in post-war environments [8].

This article summarizes the health workforce assessment conducted by the American University of Armenia's (AUA) Center for Health Services Research and Development (CHSR). This effort was the first of its kind ever conducted in NK and the largest-scale health sector assessment conducted in NK to date.

## Case Description

### Setting and Context: NK Health System

At the time of the health workforce assessment, the NK health system contained 200 health facilities including four hospitals, four dispensaries, and three ambulatories in the capital (Stepanakert), five central regional hospitals, five village district hospitals, 16 village ambulatories, 145 obstetrical centers, and nine sanitary-epidemiological stations. The system employed 274 physicians (6 years of training) and 837 nurses (2 years of training) and feldshers (3 years of training, akin to a physician's assistant)

The NK health system retains most of its Soviet structure. Under the Soviet Union's Semashko model of health services [20,21], rural primary care was delivered through an out-patient medical facility scaled to the size of the village and its environs. A health post (staffed by a nurse with a visiting physician) served the smallest of villages. An ambulatory (staffed by a physician or feldsher served larger villages. In urban settings, a multi-specialty polyclinic provided primary care. District and central regional hospitals provided secondary care, while national level hospitals and dispensaries (specialty referral centers) provided tertiary care. Sanitary-Epidemiological Stations provided basic public health services ranging from food and water safety to immunizations and disease control to laboratory services.

The current state of NK's health system is attributed to the "inherited" deficiencies from the Soviet health care system [20,21] further aggravated by the war and subsequent blockade of all but a narrow corridor linking NK to Armenia [14]. The chronically underfunded and underutilized NK health system is characterized by: lack of community participation, lack of health promotion and disease prevention activities, inadequate infrastructure, insufficient supplies, and dysfunctional health information, communication, and transportation systems, coupled with workforce development issues such as the lack of health personnel, insufficient training and retraining of health personnel, and outdated protocols [14,15]. Informal payments and distrust of the system exacerbate the situation [14]. Consequently, the majority of people either never seek health care, or seek care at late stages of their illness, leading to declines in the health status of the population [13-15].

### Procedures

To the extent practicable, the health workforce assessment followed the community engagement principles of APEXPH (The Assessment Protocol for Excellence in Public Health) [22] and MAPP (Mobilizing for Action through Planning and Partnerships) [23], which balance objective findings and expert opinion with community values and perceived priorities.

### Focus groups and in-depth interviews

Given the limited existing data, the NK health workforce training needs assessment primarily relied on qualitative methods, which included in-depth interviews (IDI) and focus group discussions (FG) with a cross-section of system planners, health care administrators, and health workers from all service levels in NK. Healthcare administrators were recruited via snowball sampling drawing upon contacts provided by international organizations having worked previously in NK. These healthcare administrators, in turn, helped to identify a pool of healthcare workers who could participate in the interviews and focus groups.

Ten focus groups totaling 41 participants (median 4, range 2-7) were conducted with NK physicians, nurses, and feldshers. A total of 11 IDIs were conducted with health system administrators, including representatives from the Ministry of Health, the NK Feldsher Academy, and health facilities. Experienced moderators supported by trained note-takers/recorders facilitated all FGs and IDIs. The interview and focus group sessions were conducted in Armenian and Russian according to the participants' preference. In keeping with the IRB approval of the American University of Armenia, audio recordings to supplement the written session notes were made only after obtaining agreement from the participants.

Both the FG and IDI guides were developed in English, translated into Armenian, and then pre-tested and revised. The semi-structured guides sought to elicit information addressing gaps in situational knowledge pertinent to the training needs assessment. Both semi-structured guides contained about 25 items, with the FG guides more oriented toward the population's practices and providers' perceived training needs and the IDIs focused more on administrators' perspective on staffing needs, training capacity, and other workforce issues. While similar, the specific prompts varied by provider type and scope of practice. So as not to deplete the limited pool of administrators, the IDI guide was pre-tested on several administrators who worked with health-related non-governmental organizations in NK. The FG guides were pre-tested using a pool of staff from a nearby health facility not targeted for inclusion in the pool of FG participants. Minor revisions were made to better elicit the desired information. The FGs lasted approximately 60 - 90 minutes. The interviews lasted approximately 60 minutes

The facilitator and note-taker prepared a detailed report of each FG and IDI (in English). Their expanded notes accompanied the report from each session and the session transcript (in Armenian and Russian, as spoken). The report reflected a consensus translation of quotes and specific phrasing where necessary. The facilitators then prepared a preliminary analysis that identified major themes and delineated the structure of the findings. These qualitative findings were then triangulated with data from the concurrent facility assessment (i.e., current staffing levels, an inventory of past training programs, and an assessment institutional infrastructure to support training).

### Synthesis

The perspectives of providers and administrators about their training needs and priorities were then synthesized with the expert opinion of the project staff, who relied on the limited existing data, their observations, and their knowledge of similar efforts conducted in similar settings. The training options were then weighed against pragmatic concerns such as resource availability and concordance with sponsor priorities and constraints. After summarizing the data in a tabular form (Table 1), a final recommended priority was assigned. Priority ratings ranged from not recommended through low, medium, and high priority status.

**Table 1:** Criterion-based prioritization of training topics by provider type and service area

	Assessment of Importance* of training for Physicians by**						Assessment of Importance of training for Nurses & Feldshers by					
	
Training program	P	A	E	R	G	Overall	P	A	E	R	G	Overall
**Target Recipient: Out-patient providers**												

**Rural**												

First Aid	█	█	█	█	█	High	█	█	█	█	█	High
IMCI/Clinical	▄	▄	█	█	█	High	▄	█	█	█	█	High
ADM/Clinical	▄	▄	█	█	█	High	▄	█	█	█	█	High
Patient Counseling	█	▄	█	█	▄	High	▄	▄	█	█	▄	Medium
IMCI/Referral						Not						Not
ADM/Referral						Not						Not
Clinical specialty						Not						Not
Facility Management						Not						Not
Health Ed Materials	█	█	█	█	█	High	▄	█	█	█	█	High
IMCI (Community)						Not						Not
ADM (Community)						Not						Not

**Regional**												

First Aid	▄	▄	█	█	█	High	█	█	█	█	█	High
IMCI/Clinical	▄	▄	█	█	█	High	█	█	█	█	█	High
ADM/Clinical	▄	▄	█	█	█	High	█	█	█	█	█	High
Patient Counseling	▄	▄	█	█	▄	Medium	▄	▄	█	█	▄	Medium
IMCI/Referral	▄	**_**	█	**_**	█	Medium	**_**	**_**	█	**_**	█	Medium
ADM/Referral						Not	**_**	**_**	█	**_**	█	Medium
Clinical specialty	█	**_**	**_**	**_**	**_**	Low						Not
Facility Management						Not						Not
Health Ed Materials	█	█	█	█	█	High	█	█	█	█	█	High
IMCI (Community)						Not						Not
ADM (Community)						Not						Not

**National**												

First Aid	▄	▄	█	█	█	High	▄	▄	█	█	█	High
IMCI/Clinical	▄	▄	█	█	█	High	▄	▄	█	█	█	High
ADM/Clinical	▄	▄	█	█	█	High	▄	▄	█	█	█	High
Patient Counseling	▄	▄	█	█	▄	Medium	▄	▄	█	█	▄	Medium
IMCI/Referral	**_**	**_**	█	**_**	█	Medium	**_**	**_**	█	**_**	█	Medium
ADM/Referral	**_**	**_**	█	**_**	█	Medium						Not
Clinical specialty	█	**_**	**_**	**_**	**_**	Low						Not
Facility Management						Not						Not
Health Ed Materials	▄	▄	█	█	█	High	▄	▄	█	█	█	High
IMCI (Community)						Not						Not
ADM (Community)						Not						Not

**Target recipient: In-patient providers**												

**Regional**												

First Aid						Not	█	█	█	█	█	High
IMCI/Clinical	**_**	**_**	▄	█	█	Medium						Not
ADM/Clinical	**_**	**_**	▄	█	█	Medium						Not
Patient Counseling	**_**	**_**	▄	█	█	Medium						Not
IMCI/Referral						Not	**_**	**_**	█	**_**	█	Medium
ADM/Referral						Not	**_**	**_**	█	**_**	█	Medium
Clinical specialty	█	▄	**_**	**_**	**_**	Medium						Not
Facility Management						Not						Not
Health Ed Materials	▄	▄	█	█	█	High						Not
IMCI (Community)						Not						Not
ADM (Community)						Not						Not

**National**												

First Aid						Not						Not
IMCI/Clinical						Not						Not
ADM/Clinical						Not						Not
Patient Counseling						Not						Not
IMCI/Referral	**_**	**_**	▄	**_**	█	Medium	**_**	**_**	█	**_**	█	Medium
ADM/Referral	**_**	**_**	▄	**_**	█	Medium	**_**	**_**	█	**_**	█	Medium
Clinical specialty	█	█	**_**	**_**	**_**	Medium						Not
Facility Management						Not						Not
Health Ed Materials						Not						Not
IMCI (Community)						Not						Not
ADM (Community)						Not						Not

**Target Recipient: Health Care Facility Administrators**												

	**Regional**						**National**					
Facility Management	█	█	█	▄	█	High						Not
Health System Mgmt						Not	█	█	█	▄	█	High

**Target recipient: Sanitary-Epidemiological Station Staff**												

	**Regional**						**National**					
Epidemiology			█	█	▄	High			█	█	▄	High
Equipment		█	**_**	▄	**_**	Medium		█	**_**	▄	**_**	Medium

**Target recipient: Community**												

IMCI (Community)	▄	▄	▄	▄	█	Medium						
ADM (Community)	▄	▄	▄	▄	█	Medium						

*Proportion of filled cell corresponds to level of importance: █ = high; ▄ = moderate; _ = low; (empty) = none

**P = Providers; A = Administrators; E = Experts; R = Resources; G = Goals

### Findings

Discussions with the various stakeholders across the service and organizational levels of the NK health system yielded rich data on the current situation, perceived challenges and needs, and priorities for intervention. Across the region, levels of healthcare facilities and training varied and perceived needs were naturally more focused on and relevant to a given person's training and role within the larger system. However, key themes and considerations emphasizing the primary care delivery system emerged and the main ideas are summarized in Table 1.

Table 1 visually depicts the training priorities as perceived by the various stakeholder groups and programmatic constraints (the columns) for a specific target group and training topic (the rows) and the resulting overall assessment. The table is organized by targeted training recipient (in-patient provider, outpatient provider, facility/system administrator, sanitary-epidemiological staff, and community) and by echelon of care (rural, regional, or national). The upper portion of the table presents recommendations for physicians and nurses operating at the same echelon of care in a side-by-side fashion. A topic perceived as not relevant or not a priority is represented by an empty cell. A thin line represents a low priority, a half-filled cell a moderate priority, and a filled cell as a high priority. The overall assessment, which represents the synthesis of all of these perspectives, but giving weight to program goals and resources constraints, is presented in words. This display allows one to compare consensus (or lack thereof) across stakeholders for a given training activity and targeted training recipient (e.g., the high degree of correlation about the need for first aid training for rural physicians), across providers operating a given level (e.g., the high degree of correlation among rural physicians and nurses/feldshers), and for a given training activity across the various echelons of the health delivery system (e.g., the inconsistent valuing of first aid training across provider setting).

### Focus Groups and In-depth Interviews

#### System level

According to system administrators, NK requires physicians to possess a medical degree and have completed a one-year internship in order to practice medicine as a therapeut (general primary care physician). Specialists require an additional clinical residency that typically lasts several years. In 1998 a licensure system for physicians, nurses, and feldshers was implemented, paralleling the system adopted in Armenia [24]. As in Armenia, the system was not sustained. Systems for delivering and tracking refresher training/continuous professional education courses never developed. Most licenses have since expired and continuing education requirements are not enforced.

Based on the size of the population being served, staffing and service levels are below expectations. Furthermore, a significant proportion of providers, especially those serving remote areas, are nearing retirement age, with little hope of replacement in the short term.

Since the cease-fire, the quality of medical education available in NK has suffered, and only a few training programs have been conducted, most by international organizations as part of their targeted humanitarian efforts. The major organizations working in NK during this period included Family Care Foundation, International Committee of the Red Cross , and Medecins Sans Frontieres. Their training programs had primarily focused on reproductive health, Integrated Management of Childhood Illnesses (IMCI), Adult Disease Management (ADM), and TB control.

Participants perceived the trainings as helpful and of high quality, stimulating demand for further training. The trainees appreciated that the trainings were free of charge and encouraged by their employers. System administrators, however, noted several shortcomings, including the lack of adaptation to local needs, protocols, and expectations; the lack of "hands-on" training components; and the provision of training without ensuring the corresponding support (e.g., the medications and equipment) needed to implement the training. Both trainees and administrators noted that these trainings had mostly targeted primary health care workers, but felt that providers at secondary and tertiary facilities also would benefit from these trainings. Furthermore, none of these sponsor-driven programs had covered the entire system, leading to imbalances in the quality of care and scope of practice, both perceived and actual, across the system. Thus, some regions within NK had received several trainings and others none, leaving a patchwork of knowledge, skill, and resources, with some providers feeling overlooked.

While emphasizing the needs in rural areas, providers stressed that all population groups would benefit from having well-trained doctors, citing the centrality of physicians in the organization and delivery of healthcare services. The head of the Republican San-Epi Station stressed the need for his staff to receive training in epidemiology, hygiene, pediatrics, and general therapy. He felt that training topics should emphasize knowledge and skills for both infectious disease surveillance and immunization system management. At the regional san-epi stations, staff felt they would benefit from trainings on general hygiene, epidemiology, parasitology, and bacteriology. System administrators noted the lack of up-to-date knowledge and skills among the entire health workforce, the lack of functional equipment, and poor conditions in general. System administrators also emphasized that the government's newly adopted decentralized management structure created a need for health financing, personnel management, planning, and leadership training for facility managers. System planners suggested coronary heart disease, hypertension, diabetes, family planning/contraception use, smoking/substance abuse, adult psychological health, nutrition, and STI/AIDS as the focal points for future training programs. They stressed, however that, although the primary care sector was important, the secondary and tertiary levels had been neglected and therefore had more training deficits. Furthermore, the planners noted that many patients now wait until their condition is severe and enter the system directly at a tertiary care site.

#### Primary care (local) facilities

Physicians and administrators from rural primary care facilities stressed the need for expanding the scope of practice of primary care physicians and the cross-training of other mid-level staff, who often were forced to address more complex cases due to patient difficulty in accessing a secondary or tertiary care center. Physicians tended to focus on the need for more specialized trainings rather than on primary and preventive services. Despite the lack of basic equipment, supplies, and laboratory reagents, most physicians believed that they were able to provide appropriate and adequate care to patients using their current skills, intuition, and experience. Most physicians believed that nurses and feldshers, however, would most benefit from primary care and preventive services training.

Many of the rural nurses and feldshers had received one or more of the recent trainings from international organizations. Nurses expressed the need for trainings related to providing and supporting primary and preventive services, but emphasized the need for suitable work conditions and stable drug supplies that would enable them to apply their new knowledge and skills in practice. Several nurses stated that they were not confident in their ability to provide adequate care when a physician is not present: only in critical situations would they rely on their own knowledge and experience.

A technical assessment of health care facilities conducted in parallel with this assessment [25] corroborated these findings, noting that most rural staff were in need of training on first aid, breastfeeding, diarrheal disease prevention and management, acute respiratory infections, STIs, reproductive health, IMCI and ADM, tuberculosis control, patient counseling, and health care management. The specific numbers of staff needing these trainings also were recorded.

### Secondary care (regional) facilities

Facility administrators from regional hospitals stated that their staff needed training in many specialty areas. This view was shared by the physicians, who added that nurses needed further specialized training as well as cross-training as nurses in secondary facilities were expected to cover multiple departments (i.e., both surgery and pediatric departments). Nurses and feldshers from regional-level facilities identified the need for training to provide and support primary and preventive services. The parallel facility assessment [25] identified first aid, breastfeeding, diarrheal diseases, acute respiratory infections, STIs, reproductive health, tuberculosis, patient counseling, and health care management, and, for those not already trained, IMCI and ADM as priority topics.

#### Tertiary care (national) facilities

Health care administrators and physicians from referral-level facilities prioritized the need for specialty training. While the training topics aligned with the major sources of morbidity and mortality, special emphasis was placed on mental health as an important concern for this post-war/frozen conflict situation.

#### Mode of training delivery

Virtually all respondents preferred trainings that emphasized active learning strategies such as interactive workshops, on-the-job training, and other practice-based trainings. Physicians preferred trainings that would last from several weeks to one to two months and combine theoretical information with practical experience in health care facilities. Several respondents suggested that international experts or specialists from Armenia could train NK specialists to become trainers for the rest of the health workforce. Physicians identified the NK capital city of Stepanakert or Yerevan (Armenia) to be the optimal setting for training.

Nurses and feldshers felt they would benefit most from trainings lasting from several days to 1-2 weeks, with regional healthcare facilities as the most suitable place for conducting their training sessions. Such an arrangement would minimize disruption of care in their communities where only a few providers operated. They believed that international specialists, as well as local specialists trained by international or Armenian experts, were best suited to deliver their training. Several nurses stressed that seasonal factors should be taken into account when planning the appropriate timing for trainings, as many health care workers from rural and regional facilities are involved in subsistence agriculture and that winter often makes travel difficult.

## Discussion and Evaluation

Based on the above information, priority training areas were identified. The determination of priorities involved consideration of several elements, including: health providers' and health system staff's assessment, the expert opinion of the project staff, the objectives and scope of the sponsor-funded project (focused on revitalizing primary and preventive health services in NK), and the availability of resources to conduct the trainings. Due consideration was given to the administrators' insistence that the training program needed to be locally relevant and hands-on. Further consideration was given to the likelihood of support from the professional and lay communities.

Effectively managing human resources first requires that the profile and professional needs of the local health care workforce be captured and considered [26]. The adaptation of training materials and methods to the local context and local needs is critically important to the success of such training programs. Adjustments must reflect the technical capabilities of local clinics and locally available and sustainable consumable supplies. Hands-on, practical training using locally sustainable resources in locally relevant contexts is essential to developing and reinforcing skills training [27] Poor adaptation may lead to the limited application of the learned skills in practice and lower satisfaction among trainees [27]. Furthermore, those who have remained in NK despite the conflict represent a largely homogenous population with strong ties to and strong sense of the community. This heightened sense of social cohesion and collective support among those remaining in NK would likely increase the uptake of trainings the participants deem valuable to the community.

• In sum, five key principles for planning training strategies were applied Trainings needed to be consistent with existing protocols and use locally attainable and sustainable supplies.

• Trainings needed to be coordinated with on-going facility renovations and refurbishments to ensure that the requisite basic primary care equipment was in place so that providers could practice the skills as taught to them.

• Trainings needed to develop a cadre of master trainers who could institutionalize the training within existing structures and not be reliant upon continued outside support.

• Where diagnostic and other laboratory equipment was provided, training on its use and maintenance also needed to be provided.

• Furthermore, trainings needed to ensure equity in access to health care services across all of NK.

These principles should be broadly applicable to other frozen conflict situations.

Based on a synthesis of these assessment factors, potential training topics, training strategies, and their targeted recipients were then ranked as first priority, second priority, or excluded from further consideration. The group of excluded topics contained mostly efforts that would improve tertiary care, reform basic professional training curricula, or, while important, were outside the scope of the project. Thus, the topics that emerged as first priority items are a collection of related training projects that built upon past efforts and predominantly address the critical needs of village-level health workers. Second priority items generally relied upon the foundation established by those first priority efforts to be fully effective. These priorities are summarized in Table 2.

**Table 2:** Recommended training and support programs by priority and target recipient

Training/support topic	Target Recipient
**First priority**	

First aid and CPR (internationally recognized)	All primary health care providers*
Clinical level IMCI (new and refresher)**	All primary health care providers
Clinical level ADM (new and refresher)**	All primary health care providers
Distribute provider resources and patient education materials	All primary health care providers
Basic healthcare management skills	Regional level healthcare facility administrators

**Secondary priorities**	

Community level IMCI training	Select communities in NK (pilot)
Patient counseling skills training	All primary health care providers
Basic epidemiology/outbreak investigations	All Sanitary-Epidemiological Station staff
Development and implementation of referral level IMCI	All secondary and tertiary levels providers
Development and implementation of referral level ADM	All secondary and tertiary levels providers

CPR = cardiopulmonary resuscitation; IMCI = integrated management of childhood illnesses; ADM = adult disease management

*providers include physicians, nurses, and feldshers; **build upon training program begun by ICRC

Included among the non-training recommendations was the suggestion to distribute provider resources and patient health education handouts focused on the pressing health problems. The AUA CHSR had developed for the Armenia Social Transition Program sixteen evidence-based patient education modules in Armenian and English that were relevant to IMCI and ADM related conditions [28]. These modules (provider information, references, and patient-friendly handouts) addressed coronary heart diseases, hypertension, injury prevention, dental health, diabetes, family planning/contraception use, healthy pregnancy/breastfeeding, smoking/substance abuse, adult and child psychological health, tuberculosis, cancer prevention, healthy nutrition, STI/AIDS, respiratory illnesses prevention in adults and elderly, and child care. Experience in Armenia suggested that the materials would be well-received by providers and patients.

### Adopted Recommendations

Deliberations with the sponsor, in light of these findings and changing programmatic constraints, led to the implementation of a 5-part training program closely aligned with these priorities over the subsequent three years. Primary care providers received first aid training that resulted in internationally recognized Red Cross certification. Primary care providers not previously trained in ADM or ICMI received an updated version of those training programs. Primary care providers were trained in basic patient counseling and health promotion skills. They were given sets of provider and patient level educational handouts and the means to make additional copies as needed (a CD-ROM containing masters of the materials was provided to each facility). Over 500 volunteers from 40 pilot communities (8 from each NK region) were trained in community-level IMCI.

The training programs utilized a train-the-trainers approach whereby international experts worked alongside a cadre of local trainers to deliver the training programs in the local languages and to assure the competence of a critical mass of local trainers to sustain the training after the completion of the project. In a clinical review of educating the medical professional, Kaufman [29] enumerates seven guiding principles for teaching practice that are reflected in the recommendations made for NK. Among these principles are engaging the learner as a contributor, building on the learner's existing knowledge and experience base, relating learning to real-life situations, and use of role models and reflection on practice [29]. These recommended training programs also were consistent with best practices and existing protocols, taking into account the supplies and medications that were locally available.

## Conclusions

Health care in post-war situations where the system's human and fixed capital are depleted is challenging enough. The addition of a frozen conflict situation, where international recognition of boundaries and authorities are lacking, introduces further complexities with healthcare planning, international aide, and funding. Despite these challenges, the precepts of evidence-based public health practice and community engagement, can contribute to meaningful assessments and determinations of priorities that balance objective needs, consensus needs, and disparate stakeholder priorities and concerns against an ambiguous political status and consequent sponsor constraints in cases such as Nagorno Karabagh.

This comprehensive workforce training needs assessment was the first of its kind in NK. The information obtained from both qualitative interviews and the facility assessment confirmed that NK health personnel, at all levels of care, were in dire need of training. Health administrators at the system and regional levels needed management and leadership training to cope with a newly decentralized and underfunded system. Hospital-based physicians desired continuing medical education in their specialty. Primary care physicians working at rural and regional level facilities desired cross-training to cope with the diverse patient population they now encountered. Nurses and feldshers reported needing broader training in primary care and preventive services and the skills to more effectively practice quasi-independently. Natural differences in priorities emerged between specialist, primary care providers, and system planners, reflective of their training, experience, and their perspective of what would be best for the health system in general versus their specific and immediate needs. Overall, the training topics mapped with the dominant current and emergent health issues facing the NK population and the desire to improve basic practice skills.

The methodology used to collect information and the criteria used to evaluate training priorities drew upon the principles and precepts of evidence-based practice [30] and community engagement [31]. Information was collected from all stakeholders within the health system and triangulated with other, objective, sources of data such as on-site facility inspections and health system surveillance and utilization data [25]. Furthermore, the assessment was conducted outside of the existing health system leadership, increasing the likelihood that participants were not trying to portray the situation in a positive light. Thus, stakeholders, sponsors, and other interested parties perceived the resulting recommendations as a fair and reasonable response to a protracted humanitarian crisis that did not exacerbate the on-going frozen conflict. This approach should be broadly applicable to other frozen conflict situations, providing an acceptable path to sustainably meeting urgent humanitarian needs without exacerbating the underlying conflict. As the US State Department and USAID noted in its 2004-2009 Strategic Plan, "Timely and effective [humanitarian] intervention minimizes suffering, contains the crisis, reestablishes local government structures that provide lasting protection, and helps lay the foundation for sustainable development" (p.28) [32].

The project focused on primary health care training of the existing workforce. Therefore, some information obtained during the assessment ultimately was beyond the scope of activities that could be implemented within this grant program. Still, the data should be of value to others contemplating programmatic efforts in NK. Not fully addressed by this analysis is the need for specialty training for secondary and tertiary level providers and the refurbishment of their facilities, the need for a comprehensive curriculum review of the Feldsher Academy programs, and the longer-term need for a workforce development plan that ensures a sufficient number of qualified providers are available to sustain the health system. Hopefully, such information will not be ignored, and can serve as a basis for efforts by others.

Frozen conflict, low resource settings are characterized by virtually collapsed health systems, disruptions to most economic sectors, and diversion of resources and personnel to defense, and weakened government capacity [7,8]. Programming responsive to both the evidence-base and to stakeholder priorities is always desirable. In these situations, such an approach is critical to balancing sponsor concerns and constraints with the community's immediate humanitarian needs while providing a foundation for long-term planning, response, and, ultimately, a seamless transition in emphasis to sustainable development [5,33].

## Competing interests

The authors declare that they have no competing interests.

## Authors' contributions

MET wrote the initial proposal, planned the conceptual approach to implementing the study, oversaw its implementation and led the analysis and interpretation.

AHD contributed to the design, planning, and implementation of the needs assessments, provided expert opinion, and contributed to the analysis.

TLH contributed to the planning of the study, conducted focus groups and interviews, and otherwise managed data collection and translation, and contributed to the analysis.

All authors contributed to the preparation of the manuscript.

## Author's information

At the time of this study, MET and TLH were with the American University of Armenia Center for Health Services Research and Development (CHSR): MET was CHSR Director and TLH was Senior Program Manager/Monitoring & Evaluation Specialist.
